# Research progress on optimization of *in vitro* isolation, cultivation and preservation methods of dental pulp stem cells for clinical application

**DOI:** 10.3389/fbioe.2024.1305614

**Published:** 2024-04-03

**Authors:** Xinxin Wang, Fenyao Li, Shuting Wu, Wenbo Xing, Jiao Fu, Ruoxuan Wang, Yan He

**Affiliations:** ^1^ Institute of Regenerative and Translational Medicine, Tianyou Hospital, Wuhan University of Science and Technology, Wuhan, China; ^2^ First Clinical College of the Ministry of Medicine, Wuhan University of Science and Technology, Wuhan, China; ^3^ Hubei Province Key Laboratory of Oral and Maxillofacial Development and Regeneration, Wuhan, China; ^4^ Department of Oral and Maxillofacial Surgery, Massachusetts General Hospital, Harvard Medical School, Boston, MA, United States

**Keywords:** dental pulp stem cells, serum-free culture, 3D cell culture, cryopreservation, stem cell property, clinical application

## Abstract

Due to high proliferative capacity, multipotent differentiation, immunomodulatory abilities, and lack of ethical concerns, dental pulp stem cells (DPSCs) are promising candidates for clinical application. Currently, clinical research on DPSCs is in its early stages. The reason for the failure to obtain clinically effective results may be problems with the production process of DPSCs. Due to the different preparation methods and reagent formulations of DPSCs, cell characteristics may be affected and lead to inconsistent experimental results. Preparation of clinical-grade DPSCs is far from ready. To achieve clinical application, it is essential to transit the manufacturing of stem cells from laboratory grade to clinical grade. This review compares and analyzes experimental data on optimizing the preparation methods of DPSCs from extraction to resuscitation, including research articles, invention patents and clinical trials. The advantages and disadvantages of various methods and potential clinical applications are discussed, and factors that could improve the quality of DPSCs for clinical application are proposed. The aim is to summarize the current manufacture of DPSCs in the establishment of a standardized, reliable, safe, and economic method for future preparation of clinical-grade cell products.

## 1 Introduction

DPSCs are derived from the pulp tissue of extracted teeth and have high proliferation, multilineage differentiation capabilities, lower immunogenicity, and immunomodulatory properties compared with other mesenchymal stem cells (MSCs) (Gronthos et al., 2000; Li et al., 2014). Additionally, human-derived DPSCs can be easily isolated from extracted teeth without causing secondary injury, and there are low ethical or legal concerns regarding their therapeutic and medical uses (Bhandi et al., 2021). As a result, both academics and medical professionals are becoming increasingly interested in studying DPSCs (Huang et al., 2009; Egusa et al., 2012). Increasing evidence shows that DPSCs are a highly effective source of MSCs and are promising candidate cells for autologous stem cell therapy in the future (Xiao and Nasu, 2014).

Although the potential therapeutic benefits of DPSCs have been demonstrated in cardiovascular, neural, bone, cartilage, and immune systems in recent years, further research and clinical trials are necessary before DPSCs can be used for disease treatment. According to a 2020 review of clinical trials, only 10 clinical trials using dental pulp-derived cells were found, and they were all used for bone regeneration or dental pulp regeneration (Yamada et al., 2020). A subsequent clinical trial in Japan studied the effectiveness and safety of a single intravenous injection of human DPSCs products in the treatment of ischemic stroke within 48 h of symptom onset (Suda et al., 2022). We searched and summarized the clinical trials using odontogenic stem cells to treat diseases so far through clinicaltrials.gov and the International Clinical Trials Registry Platform. It can be seen that the number of clinical trials has increased significantly. Although most clinical trials are for the treatment of oral diseases, there are also active attempts to treat other diseases (Tables 1, 2). Although clinical research on DPSCs is still in its initial stages, judging from a large number of clinical trials of other stem cells, DPSCs are still very promising for clinical treatment (Horwitz et al., 2002; Clé et al., 2015; Suda et al., 2020).

**TABLE 1:** Summary of the number of clinical trials of odontogenic stem cells for disease treatment registered on clinicaltrials.gov.

Diseases	Number of clinical trials	NCT number
Oral diseases (periodontitis, endodontics, tooth loss, etc.)	11	NCT03613090, NCT04983225, NCT04641533, NCT02523651, NCT03386877, NCT03451435, NCT05599087, NCT02731586, NCT05728346, NCT01814436, NCT05924373
Corona Virus Disease 2019	2	NCT04336254, NCT04302519
Cleft lip and palate	1	NCT03766217, NCT01932164
Knee osteoarthritis	1	NCT04130100
Type 1 diabetes	1	NCT03912480
Huntington disease	1	NCT06097780
Acute ischemic stroke	1	NCT04608838
Depression	1	NCT05127369

Note: The list of clinical trials included in this table is not the product of a systematic search but is filtered for relevance to the subject.

**TABLE 2:** Summary of the number of clinical trials of odontogenic stem cells for disease treatment registered on the International Clinical Trials Registry Platform.

Diseases	Number of clinical trials	Trial ID
Oral diseases (periodontitis, endodontics, atrophy of alveolar bone, etc.)	15	ChiCTR2300073144, JPRN-jRCTb040230035, NCT05924373, ChiCTR2100051466, ChiCTR2100049178, NCT04983225, NCT04641533, CTRI/2020/01/022892, NCT03386877, ISRCTN12831118, NCT02731586, NCT02523651, JPRN-UMIN000016515, NCT01814436, JPRN-UMIN000009441
Corona Virus Disease 2019	5	IRCT20140911019125N8, IRCT20140911019125N6, NCT04336254, ChiCTR2000031319, NCT04302519
Cleft lip and palate	2	RBR-3pf767, NCT03766217
Knee osteoarthritis	1	NCT04130100
Type 2 diabetes	1	ChiCTR1800018009
Lung contusion	1	IRCT20101220005426N12

Note: The list of clinical trials included in this table is not the product of a systematic search but is filtered for relevance to the subject.

To provide a satisfactory amount of cells for clinical application, DPSCs should be expanded on a large scale while maintaining their functional properties, and it must be demonstrated that DPSCs are safe and effective to be fully functional in humans. About 1–2 × 10^5^ DPSCs can be obtained from a single adult tooth (Gronthos et al., 2011), whereas to achieve desired therapeutic effect 1–10 × 10^9^ stem cells per patient were recommended in clinical application (Migliaccio et al., 2012). Two clinical trials using primary DPSCs that were not expanded *in vitro* have not observed valid results. Barbier et al. (2018) evaluated the efficacy of autologous primary DPSCs delivered in collagen matrix on alveolar healing after tooth extraction. The measurement results of computed tomography and an advanced display platform showed that there was no significant difference in the degree of bone repair between the control group and the experimental group. A clinical trial in 2023 showed that the periodontal parameters of patients who were simultaneously inoculated with L-PRF and primary DPSCs in the extraction socket of the mandibular third molar were not significantly different from the group inoculated with L-PRF alone (Cubuk et al., 2023). It is a challenge to create such DPSCs in short periods using current culturing methods (Cheung et al., 2021). On the other hand, the medium and agents used in cell culture for basic and translational studies of MSCs may raise safety concerns when it is applied to patients. Worldwide guidelines, such as European Good Manufacturing Practice (EU GMP), currently advise the use of standardized protocols for cell isolation, storage, and growth (Ducret et al., 2015a). However, because most stem cell-based medicinal product manufacturing processes alter the biological characteristics of the cells and the quality of the products produced, they might not be completely satisfying (Menard and Tarte, 2013).

**FIGURE 1:**
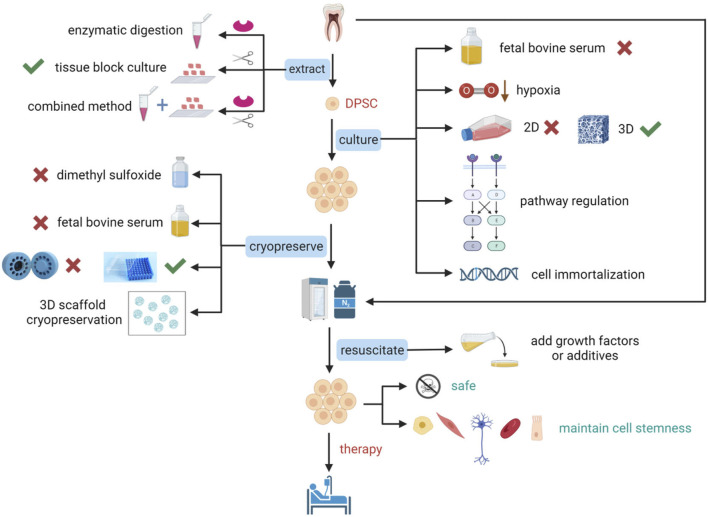
Schematic representation of optimization studies for the extraction, culture, cryopreservation, and resuscitation of DPSCs. To prepare DPSCs that conform to standards and maintain stem cell characteristics for clinical applications, optimization of many critical steps is essential. The extraction of DPSCs can be achieved using enzymatic digestion, tissue block culture, or a combination of enzymatic digestion and tissue block culture. Culture optimization of DPSCs includes not using fetal bovine serum (FBS), cultivation under low oxygen conditions, transitioning from two-dimensional (2D) culture to three-dimensional (3D) culture that is more suitable for cellular physiological environments, pathway regulation, and gene introduction for immortalization of cells. Cryopreservation optimization of DPSCs includes not using dimethyl sulfoxide (DMSO) or reducing its concentration, not using FBS, using a simpler uncontrolled-rate freezing method, freezing with a 3D scaffold, and directly freezing teeth or dental pulp tissue. Resuscitation optimization of DPSCs includes the addition of growth factors or other additives. Figure 1 was created with BioRender.com.

DPSCs undergo multiple steps from extraction to implantation, including expansion, identification, cryopreservation, and resuscitation, and each step is critical for the quality of clinical-grade DPSCs. Successful extraction of primary DPSCs from dental pulp is the prerequisite, identification of DPSCs is the guarantee, and culture and preservation without altering the biological properties of DPSCs is the key and foundation for clinical application (Figure 1). We reviewed articles and patents on optimizing methods from DPSCs extraction to resuscitation processes in the last 30 years. We analyzed the advantages and disadvantages of methodologies, and possible clinical applications, to provide new strategies for establishing standardized, reliable, safe, reproducible, and economic methods.

## 2 DPSCs extraction

### 2.1 Enzymatic digestion

In 2000, Gronthos et al. (2000) used enzymatic digestion to obtain DPSCs. Pulp tissue from the human third molar was taken and cut into small pieces in a complete culture medium. To digest the pulp tissue and separate the primary DPSCs, 3 mg/mL collagenase type I and 4 mg/mL dispase were added in a 1:1 ratio. There are a variety of digestive techniques that have been proposed for pulp separation, such as those utilized by Woods et al., who substituted neutral protease for dispase and combined type I and type II collagenases (Woods et al., 2009). Furthermore, dental pulp tissue was digested with collagenase type I at 37°C for 30–45 min by Luo et al. to prepare DPSCs (Shijie, 2020). The most popular formulation was 3 mg/mL collagenase type I and 4 mg/mL dispase (Rodas-Junco and Villicana, 2017). Although the enzymatic digestion method has several advantages, such as fast detachment, high effectiveness, and high reproducibility, its low cell adhesion rate, long digestion time, the requirement for technical expertise, and the potential for enzyme-induced cell damage have hindered its application (Ohnuma et al., 2014; Ducret et al., 2015a).

### 2.2 Tissue block culture

Couble et al. used tissue block culture to obtain DPSCs (Couble et al., 2000). They sized the pulp tissue into small pieces with sterile surgical scissors, fixed the tissue blocks on a glass slide, and cultured them under standard conditions. DPSCs crawled out of the tissue block, proliferated and migrated throughout the culture dish. Tissue block culture method is simple, safe, and does not damage cells. However, it has a low success rate, takes a longer time to obtain a large number of DPSCs, and cannot guarantee consistent experiment results.

### 2.3 Combined method

The enzymatic digestion method combined with the tissue block method is a combination of the above two methods. Using this method, rapidly growing cell populations emerged after only 1–2 days of primary culture (Raoof et al., 2014). The combination of the aforementioned culture methods provides a fast, effective, and safe cell culture technique.

Some authors believe that both enzymatic digestion and tissue block culture can be used to obtain a suitable population of DPSCs, and there is no significant difference between them (Hilkens et al., 2013; Fujii et al., 2015). The U.S. Food and Drug Administration has, nevertheless, offered suggestions for the minimal operational requirements for businesses that produce human cells, tissues, and cellular and tissue-based products. Processing that does not change the biological properties of the cells or tissues is referred to as minimal modification. Enzymatic digestion, according to the U.S. Food and Drug Administration, does not meet the regulatory criterion of minimal modification. In addition, EU GMP has requirements for the source and quality of enzymes, conditions of use, standardization of digestion procedures and verification of results. The conditions are better met by tissue block culture in contrast (Ducret et al., 2015a). However, there have been two clinical trials in Mexico and China using DPSCs extracted by enzymatic digestion and effectively promoting dental tissue regeneration (ISRCTN12831118, NCT 01814436) (Hernández-Monjaraz et al., 2018; Guo et al., 2021).

The method of extracting DPSCs has a significant impact on the efficiency of *in vitro* cultivation of DPSCs. Different extraction methods may lead to varying degrees of cell damage and a reduction in cell numbers, thereby affecting cell growth and expansion. Currently, there are relatively few research papers and invention patents focused on optimizing the primary DPSCs extraction methods. Further research is needed to develop extraction methods that meet the clinical requirements for DPSCs.

## 3 DPSCs culture medium

### 3.1 Xenogeneic serum-free culture medium

Different culture mediums can produce MSCs with different stem cell characteristics. Therefore, it is important to choose the best procedure for the proliferation of clinical-grade MSCs. The long-term culture of DPSCs has been linked to several functional changes, including reduced proliferation, altered differentiation propensity, and molecular modifications (Mehrazarin et al., 2011; Alraies et al., 2017). Fetal bovine serum (FBS) is presently added to the culture medium, which is the approach that is most frequently employed for cultivating DPSCs. FBS is a crucial additive for the *in vitro* culture of cells since it contains elements that can encourage cell adhesion, proliferation, and differentiation, including hormones, nutrients, and growth factors (Huang et al., 2009). Despite its widespread use, FBS has raised serious concerns. In addition to ethical and moral issues associated with the use of animal-derived products, its clinical use is limited because it is an extremely complex mixture containing many components of unknown composition. The use of FBS carries the risk of spreading harmful substances (such as prions, viruses, or zoonotic microorganisms) to the host (Mochizuki and Nakahara, 2018). Secondly, varying quantities of endotoxins, hemoglobin, and other hazardous components that can compromise the safety of a product may be present in serum (van der Valk et al., 2004). Additionally, there are differences in the quality and protein concentration of different batches of serum, which can affect experimental reproducibility (Thirumala et al., 2013). Furthermore, some scientists think that the inclusion of FBS proteins and peptides into cultured cells during the transplantation procedure causes the host to reject the transplanted cells (Spees et al., 2004; Gregory et al., 2006). And FBS is strictly rated by the European Medicines Agency and should be avoided for clinical applications (Wuchter et al., 2016). Therefore, the xenogeneic serum-free culture method for DPSCs is an important research hotspot.

Xenogeneic serum-free medium (XFM) is a type of serum-free medium (SFM), which refers to a medium that does not contain FBS or serum from other animal sources, with some growth factors, cytokines and other essential nutrients added. XFM has shown encouraging outcomes in many experiments that have grown DPSCs. Bakopoulou et al. (2017) found that in SFM, DPSCs had a similar isolation efficiency to that of alveolar bone marrow mesenchymal stem cells. The growth patterns of DPSCs that were passaged and grew continuously in serum-free circumstances were comparable to those in serum-containing conditions. At least in terms of cell growth, By calculating the population doubling time method to evaluate the proliferation ability of DPSCs in different culture media, it was found that DPSCs did not appear to be severely inhibited in two different SFMs. Mochizuki and Nakahara (2018) have shown that XFM for passaging culture exhibits significantly higher proliferative capacity than serum-containing medium. DPSCs maintained in XFM during long-term cultivation exhibited a normal karyotype *in vitro* and non-tumorigenic potential *in vivo*. In E8 medium, DPSCs showed decreased apoptosis and more proliferation, pluripotency, and migratory capacity, according to Xiao et al. (2018). Adipocytes, osteoblasts, neurons, and chondrocytes could all be successfully induced from DPSCs cultivated in E8 media. Created by James Thomson’s laboratory, E8 medium is a chemically defined, albumin-free medium originally used to grow induced pluripotent stem cells for use in medicine and research (Chen G. et al., 2011). Abdel Moniem et al. (2019) designed a cocktail of multiple supplements composed of recombinant human basic fibroblast growth factor (bFGF), insulin-transferrin-selenium, ascorbic acid, β-mercaptoethanol, and cholesterol. The proliferation rate of DPSCs cultured in the final cocktail was significantly increased, and the stem cell properties of the cells were not altered. This formulation is a potential and safe substitute for standard animal-derived supplements. A review by Fu et al. (Hengyi and Chenglin, 2022) summarized that there were no significant differences with/without serum on the stemness and differentiation of DPSCs. DPSCs cultured under serum-free situations exhibited typical MSCs characteristics both *in vitro* and *in vivo* and were more easily induced to undergo neurogenic differentiation and capillary-like formation. These results suggest that isolating and expanding DPSCs in serum-free conditions is a reliable and safe method.

As to the efficacy of the serum-free culture method, studies have presented conflicting conclusions. Tarle et al. (2011) indicated that SFM reduced the proliferation and differentiation ability of DPSCs compared to FBS culture. Qu et al. (2020) discovered that DPSCs exhibited a more elongated morphology in XFM, a lower proliferation rate following passaging, and a lower count of colony-forming units. In a study by Mochizuki and Nakahara, it was found that excessive proliferation and fusion of DPSCs should be avoided in XFM (Mochizuki and Nakahara, 2018). These cells may be susceptible to exogenous cellular toxicity from agents such as H_2_O_2_ and ultraviolet radiation, which can affect stem cell growth to some extent. This could be due to differences in donor characteristics, the composition of the culture media utilized, or variations in culture methods. Moreover, most of the XFM currently used in laboratories to culture DPSCs that comply with EU GMP standards are commercial products, and there are few published data to verify their effects. The feasibility and component improvement of using XFM in DPSCs culture require further extensive and in-depth research.

In recent years, several XFM patents have been disclosed for DPSCs cultivation with different compositions and purposes, as shown in Table 3. From publicly available invention patents, it can be seen that epidermal growth factor (EGF) and platelet-derived growth factor are the most commonly used growth factors, both of which promote cell proliferation and differentiation.

**TABLE 3:** Representative of patents for DPSCs cultured in XFM.

First inventor	Status	Basic culture medium	Additives	Migration	Adhesion	Proliferation	Differentiation	Other	Patent
He Han	Application	α-MEM	Insulin, porphyrin, gold nanorods, sophoridine, chlorella growth factor, progesterone, sodium selenite and glutamine	N/A	↑	↑	N/A	N/A	Han and Qianghui (2021)
Yu Kui	Application	α-MEM	EGF, glutamine, multivitamin complex, insulin, lipoic acid, propylene glycol, sodium chloride, tremella polysaccharide, extract of panax notoginseng and extract of dendrobium officinale	N/A	N/A	↑	↑	N/A	Kui et al. (2019)
Li Baojian	Application	DMEM/F12	Artemisinin, hydroxyapatite micro powder, sodium selenite, L-glutathione, biotinamide, magnolol, bFGF, zinc citrate and disodium β-glycerophosphate	N/A	↑	↑	N/A	N/A	Baojian and Bin (2022)
Wang Yifei	Application	DMEM	Serum substitute, EGF, FGF, PDGF, VEGF, L-glutamine, albumin, insulin and soy isoflavones	N/A	N/A	↑	N/A	N/A	Yifei et al. (2017)
Yu Yuansong	Application	AMMS-hMSC Medium	Serum substitute and glutamine	N/A	N/A	N/A	↑	Reduce the secretion of pro-inflammatory factor PGE2	Yuansong et al. (2023)
Zhan Zhenfeng	Application	DMEM/F12	Serum substitute, bFGF and BMP-2	N/A	N/A	↑	N/A	N/A	Zhenfeng and Qingjing (2022)
Yang Xi	Application	IMDM	SITE liquid media supplement, ascorbic acid, fibronectin, PDGF, hydrocortisone, EGF, bFGF, PTH and dexamethasone	N/A	N/A	↑	No effect	N/A	Xi et al. (2015)
Qiao Ju	Application	a-MEM	EGF, PDGF, vitamin C, TGF-β receptor inhibitor LY3200882, γ-secretase inhibitor LY411575 and DNA methyltransferase inhibitor 5-Aza-dC	↑	N/A	↑	↑	N/A	Ju et al. (2020)
Ye Qingsong	Application	a-MEM	Growth factors, hormones, proteins, vitamins, reducing substances, adhesive factors, enzyme inhibitors, trace elements and other substances	N/A	N/A	No effect	N/A	N/A	Qingsong et al. (2019b)
Zhang Xiaonan	Application	DMEM	Glucomannan, sodium alginate, plant-derived recombinant human serum albumin, sodium lactate glycinate and quercetin	N/A	↑	↑	↑	N/A	Xiaonan et al. (2020)
Guo Zikuan	Grant	α-MEM	Serotonin agonists, fibronectin, prostaglandins and citric acid or citrate salt	N/A	N/A	↑	N/A	N/A	Zikuan and Xiaofei (2021)
Mo Xiting	Application	α-MEM	Extracts of Inonotus obliquus and Ginkgo biloba leaves	N/A	N/A	↑	N/A	Not inducing spontaneous differentiation	Xiting et al. (2022)
Jiang Shu	Application	α-MEM	5%–10% human AB serum, ascorbic acid, L-glutamine, sodium penicillin and streptomycin	N/A	N/A	↑	N/A	N/A	Shu et al. (2017)
Hu Xiaodong	Application	α-MEM	Human AB serum, taurine, lipoic acid, grape seed extract, carboxymethyl chitosan, cucurbitacin, PDGF, EGF, diosgenin and choline chloride	N/A	N/A	↑	No effect	N/A	Xiaodong et al. (2018)
Zeng Haoyu	Grant	DMEM/F12	5%–20% PL, EGF, bFGF and L-alanyl-L-glutamine	N/A	N/A	↑	N/A	N/A	Haoyu et al. (2023)
Che Xiaohong	Application	MSC special basal medium	5% PL	N/A	N/A	↑	N/A	N/A	Xiaohong and Qinglin (2020)
Shi Songtao	Application	DMEM	Human PL with L-glutamine or L-glutamine analogue GlutaMAX supplement	N/A	N/A	No effect	↑	To induce T cell apoptosis, highlighting its potential in immunotherapy	Songtao (2021)
Ye Qingsong	Application	α-MEM	Human PL	↑	N/A	↑	N/A	Rich in growth factor content	Qingsong et al. (2023)

DPSCs, dental pulp stem cells; XFM, xenogeneic serum-free medium; EGF, epidermal growth factor; FGF, fibroblast growth factor; PDGF, platelet-derived growth factor; VEGF, vascular endothelial growth factor; TGF-β, transforming growth factor beta; bFGF, basic fibroblast growth factor; PGE2, prostaglandin E2; BMP-2, bone morphogenetic protein-2; PTH, parathyroid hormone; PL, platelet lysate. Note: The list of patents included in this table is not the product of a systematic search but is filtered for relevance to the subject.

### 3.2 Human blood derivatives

For the growth of stem cells, several human blood derivatives—including autologous or allogeneic human serum, human plasma, platelet lysate (PL), and its released factors—have been suggested as alternatives to FBS. Platelet-rich plasma supplementation aided in the expansion of DPSCs and preserved their stem cell characteristics (Wen et al., 2016; Saeed et al., 2017). After numerous passes, Ma et al. (2021) discovered that the expression of surface markers, chromosomal stability, and expansion rate of DPSCs grown in PL remained unchanged. These cells can influence immunological responses and could develop into neuronal, adipogenic, and osteogenic lineages. Marrazzo et al. (2016) found that low concentrations of PL (1%) could support the growth of DPSCs *in vitro* and maintain their viability. DPSCs cultured with PL had a higher rate of healing after injury and were less sensitive to the toxicity mediated by exogenous H_2_O_2_. In addition, the addition of PL was considered an appropriate strategy to promote the osteogenic and chondrogenic differentiation of DPSCs. Che et al. (Xiaohong and Qinglin, 2020) invented a method for preparing DPSCs using 5% PL and 95% basic culture medium, which achieved good results. Piva et al. (2017) found that DPSCs could be isolated and expanded to a clinical scale in an SFM containing human serum without exogenous growth factors while maintaining their characteristics of dental pulp regeneration and vascularization. Human DPSCs isolated and maintained with plasma rich in growth factors exhibited noticeably better cell stemness, according to research by Anitua et al. (2019). Growth factor-rich plasma is a platelet-rich plasma without white blood cells, which prevents inflammation from being promoted.

Human blood derivatives are considered to be safer than animal-derived products, as they do not carry the risk of animal-related diseases that could impact patient health. Moreover, human blood derivatives can be prepared through simple processes. More importantly, human blood derivatives represented by human PL are considered to comply with EU GMP requirements. However, their preparation requires a large amount of peripheral blood or cord blood, making it more expensive than animal serum. Leukocytes have been removed from human blood derivatives, but the theoretical presence of proteins and cytokines may raise concerns about immune responses, although there is no evidence that internalization of allogeneic PL proteins poses a risk of immune responses (Burnouf et al., 2016). Furthermore, the methods for extracting derivatives from human blood are not standardized, and there is no consensus so far on which specific cytokines play a major role (Astori et al., 2016). Larger animal models are needed to evaluate the therapeutic potential of MSCs cultured from human blood derivatives. Overall, human blood derivatives have several significant advantages as substitutes for animal serum in DPSCs culture. However, some limitations need to be addressed through further improvement and optimization.

## 4 Other culture conditions

The concentration of oxygen affects various cellular activities, such as cellular proliferation and differentiation. Oxygen is a crucial substrate for cellular energy production and metabolism. *In vitro*, oxygen concentrations of around 20% are used, which do not represent the physiological conditions of stem cell niches. Some studies have indicated that the oxygen content of bone marrow mesenchymal stem cells is between 1% and 7% (Peck et al., 2021). Hard dental tissue encircles the dental pulp tissue, and the only vascular pathway is the apical foramen, indicating that the oxygen partial pressure in the dental pulp tissue is also low (Yu et al., 2002). Even if there are not any precise experimental results to assess the physiological oxygen content in human dental pulp, it has been reported that rat incisor pulp has an oxygen concentration of about 3% (Yu et al., 2002). Hypoxia is a crucial factor in maintaining cell plasticity and self-renewal.

Environmental oxygen concentrations have been linked to many studies’ findings that cultured MSCs suffer from reduced proliferative rates, DNA damage, and early senescence (Estrada et al., 2012; Zayed et al., 2021). Numerous research teams have found that hypoxic culture has positive impacts on MSCs proliferation, differentiation, and apoptosis (Grayson et al., 2007; Hung et al., 2012; Kwon et al., 2017). In DPSCs, low oxygen conditions (5%) have been shown to increase proliferation rates, and stemness properties, and upregulate immune regulatory molecules (Ahmed et al., 2016; Werle et al., 2016; Zayed et al., 2021). Meng et al. (2022) discovered that hypoxia culture decreased both naturally occurring and artificially induced differentiation of human DPSCs while not affecting the morphology, phenotypic, or proliferation activity. Long-term normoxic culture can induce loss of stemness and cellular senescence in human DPSCs, while hypoxic culture could mitigate these effects. Additionally, hypoxia inhibited spontaneous and induced differentiation of human DPSCs. In a study by Zeng et al., a hypoxia-responsive long non-coding RNA was discovered to promote the proliferation and migration of human DPSCs through the PI3K/AKT signaling pathway (Zeng et al., 2022). Additionally, low oxygen environments can reduce the apoptosis rate and expression of reactive oxygen species in DPSCs, and increase colony formation ability and expression of antioxidant enzymes, thus significantly affecting the self-renewal of cells (Liu et al., 2019). Therefore, long-term hypoxic culture is advantageous for maintaining the biological characteristics of DPSCs and is suitable for large-scale expansion.

## 5 Expansion in large scale

The most popular technique for producing cell cultures in a two-dimensional (2D) system is adherent culture, in which cells are grown on surfaces. Traditional 2D culture systems are time-consuming, slow, occupy large spaces, and are prone to contamination. Furthermore, they lack the true *in vivo* cellular microenvironment and may not be suitable for large-scale culture of stem cells, which could lead to the loss of stemness and differentiation potential over time (Whitfield et al., 2013; Bara et al., 2014; Carlos Sepulveda et al., 2014). Three-dimensional (3D) cell culture can imitate the 3D cell network, the interaction between cells and the extracellular matrix (ECM), and the interaction between cells, which more closely resembles the physiological environment of the cells as compared to the 2D adherent cell culture model. This is advantageous for gene expression and signal transduction within the cells. Clustered MSCs exhibit increased levels of various pluripotency markers, such as Oct4, Sox2, and Nanog, in 3D culture, which increases their capacity for self-renewal (Bartosh et al., 2010; Guo et al., 2014; Faruqu et al., 2020; Ylostalo, 2020). Additionally, MSCs have a greater capacity to develop into bone, fat, and nerve cells (Yamaguchi et al., 2014; Petrenko et al., 2017; Song et al., 2018; Kim et al., 2021), and their therapeutic effectiveness is increased by boosting the release of anti-inflammatory cytokines and chemokines, which improves their functional properties (Cui et al., 2017; Sun et al., 2018).

**FIGURE 2:**
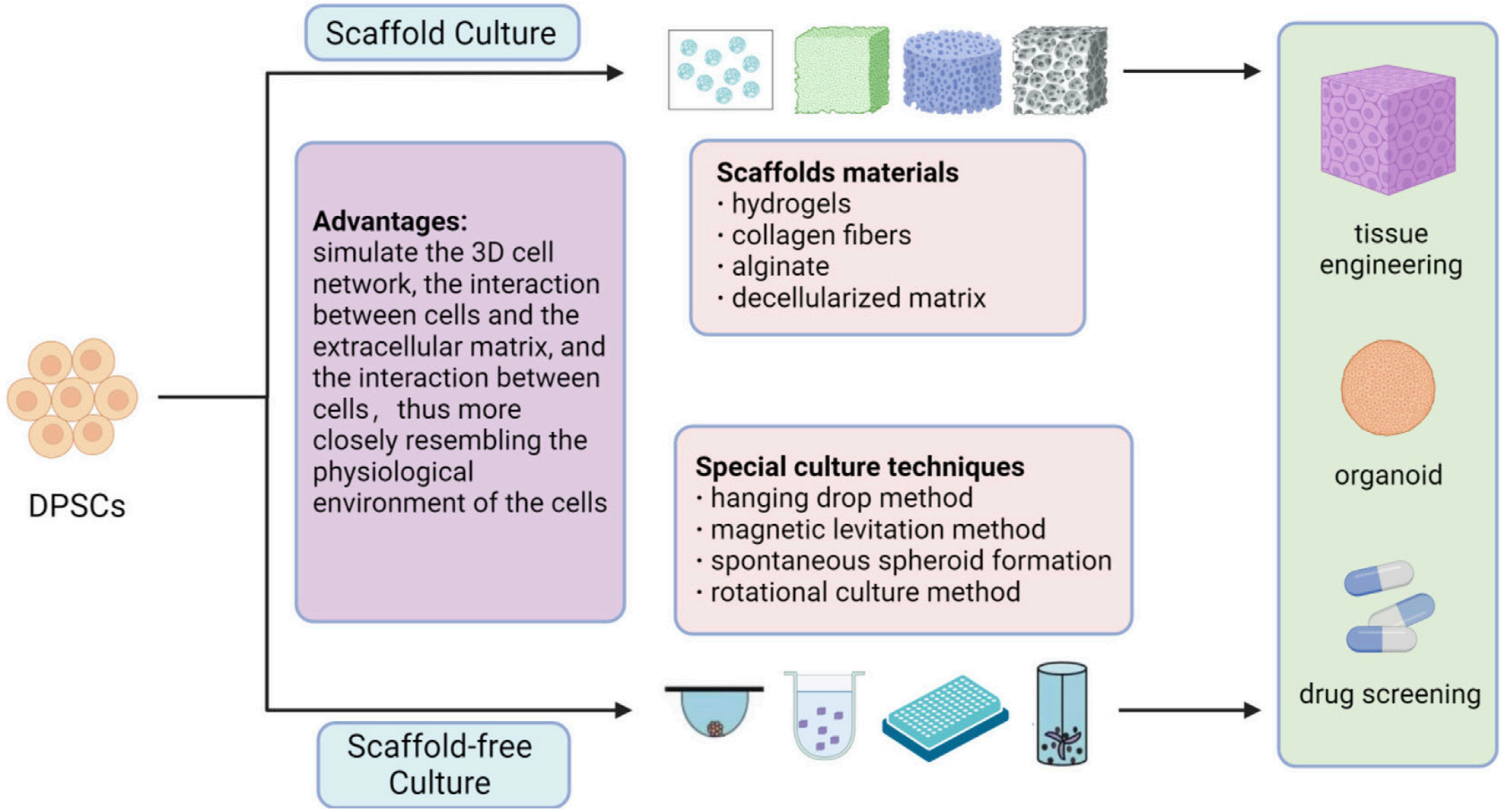
3D Culture of DPSCs. The 3D culture of DPSCs includes two methods: scaffold culture and scaffold-free culture. Scaffold culture commonly employs materials such as hydrogels, collagen fibers, alginate, and decellularized matrix. Special techniques used for scaffold-free culture include hanging drop method, magnetic levitation method, spontaneous spheroid formation, and rotational culture method. 3D cell culture can simulate the interactions between cells and the extracellular matrix, as well as cell-to-cell interactions, more closely resembling the physiological environment of cells. This is beneficial for gene expression and signal transduction within the cells and can be used for tissue engineering, organoid construction, drug screening, and other applications. Figure 2 was created with BioRender.com.

3D cell culture involves seeding cells onto a 3D scaffold or cultivating them into 3D aggregates (Chuhan et al., 2023), including scaffold-based and scaffold-free methods (Figure 2; Table 4).

**TABLE 4:** Studies of 3D culture of DPSCs.

	Scaffold classification	Scaffold materials	Results	References
Scaffold Culture	Hydrogels	0.25% peptide hydrogel	It supported good growth and normal morphology of DPSCs	Lixin et al. (2018)
		PCL and GelMA	DPSCs were uniformly distributed throughout the hydrogel components and had high cell viability and osteogenic differentiation ability	Buyuksungur et al. (2021)
	Collagen fibers	1% collagen type I	It supported good growth and normal morphology of DPSCs	Lixin et al. (2018)
		Collagen-nano-hydroxyapatite/phosphoserine	DPSCs exhibited higher activity, and had greater osteogenic differentiation ability	Salgado et al. (2020)
	Alginate	GOSA	DPSCs showed good proliferative capacity without significant cytotoxicity and were suitable for serum-free culture environment	Mansouri et al. (2021)
	Decellularized scaffolds	Decellularized ECM scaffolds derived from porcine and human adipose tissue	Human adipose tissue solid foam was a better non-calcifying soft tissue regeneration scaffold, while the porcine adipose tissue solid foam was a more suitable choice for mineralized bone regeneration	Luzuriaga et al. (2022)
		Decellularized dental matrix	It can simulate the developmental microenvironment related to odontogenesis, regenerate functional teeth after tooth avulsion due to trauma, and support the continued development of tooth roots	Guo et al. (2021)
	Others	Non-porous microcarriers: cross-linked dextran with a diameter of 190 μm; porous microcarriers: cellulose structure with a diameter of 230 μm	Both porous and non-porous microcarriers were suitable for DPSCs expansion in dynamic culture conditions	Foldes et al. (2021)
	Culture Techniques	Culture Methods	Results	References
Scaffold-Free Culture	Hanging drop method	Ultra-Low attachment culture dishes	DPSCs cultured in suspension using SFM supplemented with bFGF and EGF maintained their biological characteristics	Pisciotta et al. (2018)
		Low-attachment culture plates	The expression of tooth/bone differentiation markers in DPSCs was upregulated, and mineralized nodules were formed	Kawashima et al. (2017)
	Magnetic levitation method	Ultralow attachment plates combined magnetic nanoparticle	The trilineage differentiation ability of DPSCs was significantly enhanced, and the genes related to osteogenesis, adipogenicity, angiogenesis and stemness were significantly enhanced. MAPK and NF-kB signaling pathways were significantly activated, improving the apoptotic effect of 3D culture	Chan et al. (2021)
	Spontaneous spheroid formation	Ultra-low attachment and U-bottomed StemFit 3D plates	DPSCs had higher levels of differential gene expression in regeneration-related gene categories and had a stronger capacity for multipotent differentiation	Bu et al. (2020)
	Rotating culture method	Spinner flasks and microcarrier beads	Proliferation and lineage-specific differentiation of DPSCs were upregulated, and the expression of oncogenes and apoptosis genes in DPSCs was significantly suppressed	Wu et al. (2022)

PCL, poly(ε-caprolactone); GelMA, gelatin methacrylate; GOSA, graphene oxide/sodium alginate; ECM, extracellular matrix; 3D, three-dimensional; DPSCs, dental pulp stem cells; SFM, Serum-free medium; bFGF, basic fibroblast growth factor; EGF, epidermal growth factor. Note: The list of studies included in this table is not the product of a systematic search, but rather a screening of research papers related to the topic.

### 5.1 Scaffold culture

Scaffold culture refers to using scaffold materials in 3D space to construct a support structure for cell growth. The scaffold is typically composed of porous or fibrous structures made of polymers, and it is a more traditional approach to 3D cell culture. Scaffolds can provide support structures for cell growth, as well as appropriate growth environments and communication signals between cells. However, there may be issues with biocompatibility and interactions between cells and materials. Scaffold cultivation of DPSCs is more suitable for tissue engineering. Tissue engineering aims to construct artificial tissues and organs that are similar to autologous tissues to achieve medical treatment, and 3D cell culture is a critical step in achieving this goal. Combining MSCs with the right carriers or growth factors can significantly improve the capacity for tissue regeneration (Lee et al., 2019; Lew et al., 2019). The multipotent differentiation potential of DPSCs can be used in the tissue regeneration of various fields.

The culture effects of DPSCs using different scaffolds have been studied. Common materials used include hydrogels, collagen fibers, alginate, and decellularized matrix (Weidong and Runyuan, 2021). Buyuksungur et al. (2021) used a combination of poly(ε-caprolactone) (PCL) and gelatin methacrylate (GelMA) to create a 3D-printed hybrid scaffold loaded with DPSCs. DPSCs were uniformly distributed throughout the hydrogel components and had high cell viability and osteogenic differentiation ability. Salgado et al. (2020) found that DPSCs cultured dynamically in a collagen-nano-hydroxyapatite/phosphoserine biomimetic 3D scaffold were well distributed, exhibited higher activity, and had greater osteogenic differentiation ability. Kong et al. (Lixin et al., 2018) compared the effects of a 1% collagen type I scaffold and 0.25% peptide hydrogel scaffold on DPSCs proliferation and found that both scaffold materials supported good growth and normal morphology of DPSCs. Two clinical trials reported that DPSCs seeded onto collagen scaffolds could induce bone tissue regeneration in bone defects caused by periodontal disease after transplantation (Ferrarotti et al., 2018; Hernández-Monjaraz et al., 2018). Mansouri et al. (2021) prepared a graphene oxide/sodium alginate (GOSA) scaffold, and DPSCs cultured on the 3D porous scaffold showed good proliferative capacity without significant cytotoxicity. Moreover, the toxicity of DPSCs cultured in serum-rich conditions was stronger than in SFM on the scaffold, indicating the potential for DPSCs to be cultured on the scaffold under serum-free conditions for clinical use. Luzuriaga et al. (2022) combined human DPSCs with decellularized ECM scaffolds derived from porcine and human adipose tissue *in vitro* and processed them into 3D solid foams. The results showed that the human adipose tissue solid foam was a better non-calcifying soft tissue regeneration scaffold, while the porcine adipose tissue solid foam was a more suitable choice for mineralized bone regeneration. These decellularized ECM-derived materials can be used to create customized tissue engineering bioinks thanks to modern 3D bioprinting technologies (Dzobo et al., 2019; Kabirian and Mozafari, 2020). Guo et al. (2021) used decellularized dental matrix combined with human DPSCs aggregates to simulate the developmental microenvironment related to odontogenesis, enabling functional tooth regeneration in patients with tooth avulsion due to trauma, and supporting the continued development of human tooth roots. Foldes et al. (2021) found that cell proliferation rate was 5–10 times higher on two types of microcarriers (non-porous microcarriers: cross-linked dextran with a diameter of 190 μm; porous microcarriers: cellulose structure with a diameter of 230 μm) than those without microspheres. Both porous and non-porous microcarriers were suitable for DPSCs expansion in dynamic culture conditions. The ability to increase the culture volume in 3D space without the requirement for passaging is the use of microcarriers’ greatest benefit.

In recent years, many invention patents for 3D scaffolds used in DPSCs culture have been published, as shown in Table 5.

**TABLE 5:** Representative of patents for DPSCs 3D culture.

First inventor	Status	Scaffold and additives	Proliferation	Differentiation	Application	Patent
Zhou Yachuan	Grant	Chitosan/β-glycerophosphate hydrogel	↑	↑	Promote DPSCs proliferation and odontoblast differentiation	Yachuan et al. (2021)
Liu Jie	Application	Hydroxybutyl chitosan modified by gallic	↑	N/A	Improve the retention time and survival rate of stem cells in the body	Jie et al. (2023)
Ye Qingsong	Application	GelMA-bFGF hydrogel composite material	↑	N/A	Neural tissue engineering	Qingsong et al. (2017)
Feng Xingmei	Application	Chitosan scaffold	N/A	N/A	Improve nerve damage and functional recovery	Xingmei et al. (2017)
Zhang Jingying	Application	Chitosan, hydroxyapatite and β-tricalcium phosphate composite scaffold	N/A	↑	Bone tissue engineering	Jingying (2019)
Chen Haijia	Application	Crosslinked porcine gelatin microcarriers	N/A	N/A	Repair alveolar bone degeneration	Haijia et al. (2017)
Wang Songling	Grant	Wnt3a sustained-release oxidized alginate gel and collagen or PCL scaffold	N/A	N/A	Repair periodontal ligament and cementum defects	Songling et al. (2022)
Gao Zhenhua	Grant	Rat acellular submandibular gland matrix freeze-dried scaffold	N/A	N/A	Dental pulp tissue regeneration	Zhenhua et al. (2023)
Chen Gang	Grant	Dental pulp tissue extracellular matrix injection material	N/A	N/A	Dental pulp tissue regeneration	Gang et al. (2020)
Yelick Pamela C	Grant	GelMA hydrogel and photoinitiator	N/A	N/A	Dental pulp tissue regeneration	Yelick and Khademhosseini (2023)
Zhou Jian	Grant	Chitosan/bis-4-aldehyde benzoate polyethylene glycol ester scaffold, EGF and IL-2	N/A	N/A	Pulp and dentin regeneration	Jian et al. (2021)
Cen Lian	Application	Injectable hyaluronic acid gel scaffold and TGF-β1	N/A	N/A	Pulp and dentin regeneration	Lian et al. (2015)
Celiz Adam D	Grant	Particles or hydrogels of triacrylates and encapsulating compositions	↑	↑	Treat periodontal disease or pulp infection	Celiz et al. (2022)
Unmentioned	Application	Using a shaker device in a 3D culture environment	N/A	No effect	Tissue damage repair	Unmentioned (2019)
Wasielewski Ray C	Application	Porous matrix formed by grinding hard teeth	N/A	N/A	Various clinical uses	Wasielewski (2018)

DPSCs, dental pulp stem cells; 3D, three-dimensional; GelMA, gelatin methacrylate; bFGF, basic fibroblast growth factor; PCL, poly(ε-caprolactone); EGF, epidermal growth factor; IL-2, interleukin 2; TGF-β1, transforming growth factor beta 1. Note: The list of patents included in this table is not the product of a systematic search but is filtered for relevance to the subject.

### 5.2 Scaffold-free culture

Scaffold-free culture refers to special culture techniques that allow cells to self-organize into a 3D growth environment without the use of external scaffold materials (Breslin and O'Driscoll, 2013). This is achieved through techniques such as hanging drop method, magnetic levitation method, spontaneous spheroid formation, and rotational culture method which take advantage of cell aggregation tendencies to form aggregates or 3D microspheres. The method is more natural and closer to the *in vivo* environment, allowing for better maintenance of the self-renewal and differentiation of stem cells. Additionally, it eliminates the need for complex scaffold materials, making it simpler and faster to prepare and avoiding the disadvantages of poor scaffold degradation ability and negative effects on cell growth. For large-scale *in vitro* development of DPSCs for clinical purposes, this culture approach is more appropriate. However, due to the lack of external scaffold structures, cell growth and differentiation may be more disorderly, requiring more complex culture techniques and conditions.

Many researchers have explored the promoting effects of different types of 3D spheroid culture on the biological characteristics of DPSCs. Pisciotta et al. (2018) found that DPSCs cultured in suspension using SFM supplemented with bFGF and EGF maintained their biological characteristics. Kawashima et al. (2017) cultured DPSCs in a 3D sphere culture system similar to embryonic stem cell suspension culture. The expression of tooth/bone differentiation markers in DPSCs was upregulated, and mineralized nodules were formed, indicating that this 3D sphere culture system may be suitable for inducing hard tissue formation. Chan et al. (2021) demonstrated that DPSCs cultured as magnetic suspension 3D spheres had significantly enhanced tri-lineage differentiation ability. These cells also displayed enhanced expression of genes associated with stemness, adipogenesis, osteogenesis, and angiogenic processes. The apoptotic effects of 3D culture were also improved by activating the MAPK and NF-kB signaling pathways, significantly boosting the therapeutic impact of DPSCs. According to the research by Bu et al., DPSCs cultured in microsphere-forming plates had higher levels of differential gene expression in regeneration-related gene categories and had a stronger capacity for multipotent differentiation than DPSCs cultured in conventional monolayer plates (Bu et al., 2020). Wu et al. (2022) found that 2,3,5,40-tetrahydroxystilbene-2-O-D-glucoside may improve DPSCs stemness when used in a rotating bioreactor. Additionally, the expression of oncogenes and apoptotic genes in DPSCs was markedly decreased. Because the rotating bioreactor overcomes the limitation of surface area, it provides more space for cell yield and can be used for large-scale production and culture of human DPSCs for tissue regeneration. In addition, Itoh et al. (2018) used thermoresponsive hydrogel to create a scaffold-free 3D structure of DPSCs, which has the necessary self-organizing ability as a transplanted tissue. It can differentiate into odontoblasts *in vivo* and form a vascular-rich dental pulp-like tissue. However, there are relatively few patents related to scaffold-free 3D culture of DPSCs, which poses great difficulties for its actual clinical application.

Currently, 3D-cultured stem cells are widely used in drug screening models, the construction of human organoids, tissue engineering, and other fields, and have shown excellent performance. The 3D culture of DPSCs can be closer to its natural biological environment, which can better preserve the physiological characteristics of cells, and enable DPSCs to grow and expand better. However, the 3D culture of DPSCs requires the use of specialized culture vessels and equipment, which results in relatively high costs. Controlling cell growth and expansion in a 3D environment is more challenging, thus requiring careful monitoring and regulation of the cell growth status. Additionally, several studies have demonstrated that long-term MSCs culture in 3D culture conditions can result in decreased cell proliferation and accelerated rates of cell death because of cellular aging (Mueller-Klieser, 1997; Tsai et al., 2015; Tsai et al., 2019; Son et al., 2021). Dynamic conditions can also generate air bubbles, and cell injury has been linked to the gas-liquid interface (Walls et al., 2017). Overall, DPSCs 3D culture has significant value, but there are also technical and cost-related difficulties that require more extensive and in-depth research.

## 6 Pathway regulation

New targets for controlling stem cell fate and promoting DPSCs stemness *in vitro* can be found by investigating the signaling pathways involved in proliferation and differentiation. In DPSCs, the Wnt and Notch pathways are critical for controlling self-renewal and differentiation (Scheller et al., 2008; Uribe-Etxebarria et al., 2017). Hanna et al. (2023) found that two small molecule GSK-3 inhibitors (Wnt signaling agonists) can promote the proliferation of human DPSCs, enhance the expression of stem cell markers, and the cells did not undergo apoptosis. Uribe-Etxebarria et al. (2020) stated that simple pharmacological treatment with Wnt-3a (Wnt ligand), perhaps in the presence of FBS, maintained DPSCs for a longer period without compromising their stem cell properties due to spontaneous osteoblast differentiation *in vitro*. The research group also found that N-[N-(3,5-Difluorophenacetyl)-L-alanyl]-S-phenylglycine t-butyl ester inhibited Notch and reduced the expression of core factors in DPSCs, which decreased their ability to differentiate into fully differentiated osteoblasts and adipocytes (Uribe-Etxebarria et al., 2017). In physiologically generated DPSCs, Wnt activation resulted in a rapid rise in Jagged1 transcription levels. In DPSCs culture, this elevated Jagged1 transcription may ultimately activate the Notch receptor (Uribe-Etxebarria et al., 2017).

The SCF/c-Kit signaling pathway had also been shown to play a role in the self-renewal of human DPSCs and to maintain a long-term pool of human DPSCs (Cucco et al., 2020). Nuclear factor I-C controlled Sox2 to play a role in DPSCs cell proliferation, stem cell niche maintenance, and cell fate determination (Lee et al., 2022). bFGF regulated the self-renewal of DPSCs, maintained pluripotency, inhibited mineralization, and promoted neuronal differentiation mediated by the FGFR and PLCγ pathways (Osathanon et al., 2011). Through certain MAPKs and Smad2/3 signaling pathways, as well as the production of RUNX2, TGF-β is essential for the self-renewal and differentiation of distinct stem cell sources (Park et al., 2019). Recombinant protein factors, however, have proved costly and difficult to make. Smaller molecules that are easier to synthesize play an important role in influencing cellular behavior. Through some cell signaling pathways, including Ras-GAP, ERK1, and mTOR, small compounds can change the features of DPSCs by increasing the expression of genes related to stemness and reducing their propensity to differentiate (Al-Habib et al., 2013) (Table 6).

**TABLE 6:** Studies on pathway regulation of DPSCs with regard to biological characteristics.

First author	Molecules	Signaling pathways/Mark molecules	Results	References
Samer Hanna	GSK-3 inhibitors (Wnt signaling agonists)	Wnt pathway	DPSCs proliferative ability and expression of stem cell markers were enhanced, and the cells were not apoptotic	Hanna et al. (2023)
Verónica Uribe-Etxebarria	Wnt-3a (Wnt ligand)	Wnt pathway	DPSCs could be maintained for longer periods without compromising their stem cell properties due to spontaneous osteoblastic differentiation *in vitro*	Uribe-Etxebarria et al. (2020)
Verónica Uribe-Etxebarria	N-[N-(3,5-Difluorophenacetyl)-L-alanyl]-S-phenylglycine t-butyl ester	Notch pathway	The expression of core factors and the ability of osteogenic and adipogenic differentiation of DPSCs were reduced	Uribe-Etxebarria et al. (2017)
Carolina Cucco	N/A	SCF/c-Kit pathway	This pathway functioned in the self-renewal of human DPSCs and enabled long-term maintenance of the stem cell pool in human dental pulp	Cucco et al. (2020)
Dong-Seol Lee	Nuclear factor I-C	Sox2	It played a role in DPSCs cell proliferation, stem cell niche maintenance, and cell fate determination	Lee et al. (2022)
Thanaphum Osathanon	bFGF	FGFR and PLCγ pathways	It regulated self-renewal of DPSCs, maintained pluripotency, inhibited mineralization, and promoted neuronal differentiation	Osathanon et al. (2011)
Kyung-Ran Park	TGF-β	MAPKs and Smad2/3 pathways, RUNX2	TGF-β is essential for the self-renewal and differentiation of distinct stem cell sources	Park et al. (2019)
Mey Al-Habib	Synthetic small molecules	Pathways such as ras-gap, erk1, and mtor	Small molecules affected the properties of DPSCs by increasing the expression of genes related to stemness and reducing their differentiation propensity	Al-Habib et al. (2013)

DPSCs, dental pulp stem cells; bFGF, basic fibroblast growth factor; TGF-β, transforming growth factor beta. Note: The list of studies included in this table is not the product of a systematic search, but rather a screening of research papers related to the topic.

To fully use the potential of DPSCs, selective stimulation of signaling pathways can govern cell proliferation and improve their attributes. Pathway modulation does not require genetic manipulation and is gentler than cell reprogramming, making it a potentially ideal cellular therapy approach and opening up new possibilities for the use of DPSCs in cell therapy (Uribe-Etxebarria et al., 2023). Indeed, signal pathways are typically complex networks composed of multiple molecules, and the interactions among these molecules are highly intricate. Therefore, more extensive and in-depth research is required to enhance our understanding of signal pathways and apply them to the large-scale *in vitro* expansion of DPSCs.

## 7 Cell immortalization

Although DPSCs have a high self-renewal capacity, their lifespan is limited *in vitro* after long-term culture due to the restriction of actual division numbers, ultimately leading to irreversible proliferation arrest, namely replicative senescence, which may result in loss of their stem cell potential (Mokry et al., 2010; Alraies et al., 2017; Zeng et al., 2023). Primary human DPSCs cultured to the seventh generation will enter cell senescence (Galler et al., 2006). According to another study, DPSCs can typically reach a generational height of 13 before reaching full senescence (Wilson et al., 2015). The replicative senescence of stem cells is usually thought to be caused by genomic instability following crucial telomere shortening (Yin et al., 2016). Immortalized stem cells generated through genetic modification or gene introduction have telomere repair ability and at least 200 more divisions of proliferation ability. Compared to general DPSCs, immortalized DPSCs can proliferate indefinitely, have higher biological stability, and exhibit more consistent differentiation ability and phenotype stability.

During cell division, telomerase possesses enzymatic activity to prolong and maintain telomere length. Human telomerase reverse transcriptase (hTERT) plays a crucial role in maintaining telomere length during cell division and regulating the stemness and self-renewal characteristics of stem cells (Venturini et al., 2011; Orimoto et al., 2020b). Some studies and patents have prepared immortalized DPSCs by introducing hTERT alone or in combination with other genes. Egbuniwe et al. (2011) and Egbuniwe et al. (2013) expressed hTERT in DPSCs using a retroviral vector, and the DPSCs were able to maintain the phenotype of odontoblasts and avoid senescence, possibly due to reduced p16 expression. However, some studies suggest that overexpression of hTERT alone may not be sufficient to immortalize cells (Colgin and Reddel, 1999). When Orimoto et al. tried expressing TERT alone, they were unable to produce immortalized DPSCs; instead, they created a new immortalized DPSCs line by co-expressing a mutant version of cyclin-dependent kinase 4, cyclin D1, and TERT, which preserved the original biological properties (Orimoto et al., 2020a). Ueda et al. (Minoru, 2015) provided a method for producing immortalized DPSCs by introducing four genes (TERT, bmi-1, E6, E7). The conditioned medium of immortalized DPSCs was collected and further prepared into a pharmaceutical composition for cancer therapy containing 1.5 times more insulin-like growth factor and vascular endothelial cell proliferation factor. Furthermore, Kornsuthisopon et al. (2022) immortalized human DPSCs by SV40T antigen transfection, extending their lifespan to the 25th passage with better proliferation ability. The immortalized DPSCs were endowed with enhanced proliferation, migration, and apoptotic abilities without tumorigenicity, and they displayed the same stem cell surface markers as the original DPSCs, according to Li et al. (2020) utilization of the iggyBac transposon-mediated method to stably express SV40T-Ag. It is worth noting that the immortalization of DPSCs by this method is reversible, and the cells maintain high biological characteristics after de-immortalization (Table 7).

**TABLE 7:** Studies on immortalization of DPSCs.

First author	Introduced genes	Results	References
Obi Egbuniwe	hTERT	Immortalized DPSCs were able to maintain the phenotype of odontoblasts, avoid senescence, possibly due to reduced p16 expression	Egbuniwe et al. (2011), Egbuniwe et al. (2013)
Ai Orimoto	cyclin-dependent kinase 4, cyclin D1, and TERT	Immortalized DPSCs proliferated continuously and at a faster rate without compromising differentiation potential and signs of aging	Orimoto et al. (2020a)
Ueda Minoru	TERT, bmi-1, E6, E7	The conditioned medium of immortalized DPSCs was collected and further prepared into a pharmaceutical composition for cancer therapy containing 1.5 times more IGF-1 and VEGF	Minoru (2015)
Chatvadee Kornsuthisopon	SV40 T-antigen	The lifespan of immortalized DPSCs was extended to the 25th passage with better proliferative ability	Kornsuthisopon et al. (2022)
Xiangfen Li	SV40 T-antigen	The immortalized DPSCs were endowed with enhanced proliferation, migration, and apoptotic abilities without tumorigenicity, and they displayed the same stem cell surface markers as the original DPSCs. The immortalization of DPSCs prepared by this method is reversible, and the cells still maintain high biological characteristics after de-immortalization	Li et al. (2020)

hTERT, human telomerase reverse transcriptase; DPSCs, dental pulp stem cells; IGF, insulin-like growth factor; VEGF, vascular endothelial growth factor. Note: The list of studies included in this table is not the product of a systematic search, but rather a screening of research papers related to the topic.

Immortalized DPSCs can maintain stable proliferation for a long time *in vitro*, which is useful for long-term research and applications. However, cell immortalization often requires the use of chemical drugs or gene technologies, which may raise ethical concerns. Although some researchers have reported that hTERT-immortalized DPSC lines do not exhibit tumorigenic potential in the short term (Wilson et al., 2015; Inada et al., 2019; Li et al., 2020), long-term safety concerns such as genetic variation and tumorigenesis require continuous monitoring and control. There is a dearth of thorough descriptions of their biological behavior, and *in vivo*, applicability is still somewhat restricted (Li et al., 2020). Moreover, when immortalized cells gain strong proliferative ability, their differentiation capacity is greatly reduced (Wang et al., 2019; Li et al., 2020). In addition, the clinical effectiveness of Immortalized DPSCs needs to be verified through sufficient clinical trials, and the problems of large-scale production and storage need to be solved. These issues limit the current application of immortalized DPSCs, but they are expected to be resolved in the future.

## 8 Cryopreservation of DPSCs

Due to the temporal discrepancy between the donor and recipient in stem cell transplantation, it is difficult to ensure that the cells can be used immediately. Long-term *in vitro* culture of stem cells can lead to the introduction of genetic or epigenetic instability, which may alter their characteristics and functional properties, such as reduced differentiation capacity, senescence, and apoptosis, thereby affecting clinical benefits (Safwani et al., 2012; Moll et al., 2014; Turinetto et al., 2016). In addition, long-term *in vitro* culture is time-consuming and expensive, which highlights the importance of cryopreservation of stem cells. Cryopreservation techniques must be effective and reliable for stem cell-based therapies to be used successfully in clinical settings. Currently, most of the MSCs used clinically have been cultured *in vitro* and stored at low temperatures.

**FIGURE 3:**
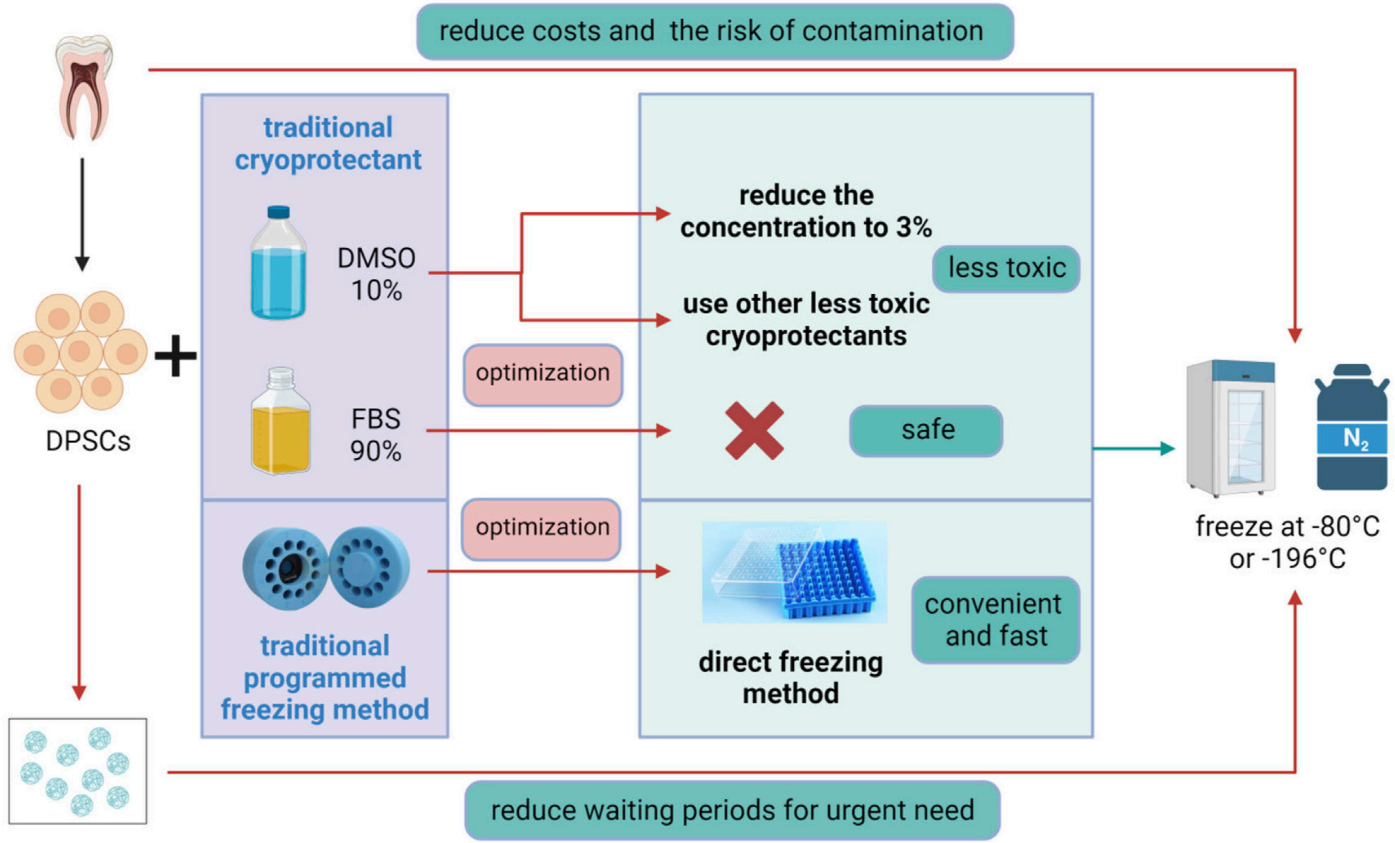
Optimization of DPSCs Cryopreservation. The traditional method for DPSCs cryopreservation involves programmed freezing in the presence of a cryoprotectant, usually, dimethyl sulfoxide (DMSO), combined with fetal bovine serum (FBS). Optimization methods include reducing the concentration of DMSO to 3% or replacing it with other low-toxicity cryoprotectants, avoiding the use of hazardous FBS, and adopting a convenient and rapid direct freezing method, followed by storage at −80°C or in liquid nitrogen at −196°C. In addition, 3D scaffold cryopreservation can eliminate the long waiting period for patients with acute diseases, while direct cryopreservation of teeth or dental pulp tissue can reduce costs and the risk of tissue contamination. Figure 3 was created with BioRender.com.

The cryopreservation process of cells includes dissolving a certain number of cells in cryopreservation solution, aliquoting them into cryovials, and then placing the aliquoted cryovials into −80°C or liquid nitrogen tanks for freezing. A good cryopreservation strategy should be able to overcome problems after thawing, such as poor cell recovery, reduced cell viability, and inhibited cell proliferation. Existing cryopreservation methods can maintain cells for long-term preservation without changing their stemness (Conde et al., 2016; Raik et al., 2020). The standard method for laboratory-based MSCs cryopreservation involves moderate freezing while a cryoprotectant and FBS are present to shield cells against accelerated freezing and thawing cycles (Hanley et al., 2013; Duan et al., 2018; Pilbauerova and Suchanek, 2018). However, this method is an improvement of the cryopreservation method for hematopoietic stem cells and lymphocytes and is not the optimal method for cryopreserving MSCs (Sharma et al., 2014). The optimization of stem cell cryopreservation mainly comes from the challenges of two aspects: ①whether the composition of the cryoprotectants will reduce the vitality and function of stem cells after recovery; ②safety issues (Figure 3).

### 8.1 Cryoprotectants and FBS

Currently, the most commonly used cryopreservation solution for odontogenic stem cells in laboratories is a mixture of dimethyl sulfoxide (DMSO) and FBS (with or without basal culture medium) (Conde et al., 2016; Pilbauerova and Suchanek, 2018). DMSO, the best permeable cell cryoprotectant at the moment, can guard against harm from intracellular ice crystal formation, osmotic pressure fluctuations, and cell structural issues during deep cryopreservation. Cell cryopreservation uses cryoprotectants to lower the freezing point. Cryoprotectants are compounds added to the freezing medium to prevent cell damage during cryopreservation, and they are essential reagents in almost all cell cryopreservation protocols. The effectiveness and stability of cryopreserved stem cell products mainly depend on the cryoprotectants. However, DMSO is also a hazardous chemical reagent that is mostly used in cell recovery and culture operations (Nishigaki et al., 2011), which can impair the ability of frozen MSCs to proliferate and differentiate (Lindemann et al., 2014). Moreover, DMSO, being a “universal solvent,” possesses strong skin penetration and volatilization characteristics, which can pose a risk to the health of operators.

Numerous strategies have been explored to reduce the toxicity of cryoprotectants and minimize cryo-damage, including attempts to lower the concentration of DMSO and utilize other low-toxicity cryoprotectants. Demirci et al. (2014) found that preserving DPSCs in a solution containing 20 μg/mL of borate and 5% DMSO can improve their survival rate. The solution also has no impact on the expression of surface antigens or the ability to differentiate into bone and cartilage. Adding high molecular weight hyaluronic acid to the cryoprotectant can reduce the concentration of DMSO from 10% to 3% without affecting cell viability or MSCs markers, and the proliferation of DPSCs is increased (Pilbauerova et al., 2022). With a magnetic field freezing protocol, the amount of DMSO can be reduced to 3% (Lee et al., 2012a). The presence of a steady magnetic field enhances the effectiveness of DMSO-free DPSCs cryopreservation (Lin et al., 2015). Furthermore, studies have shown that DPSCs cryopreserved with 10% glycerol and 10% ethylene glycol have similar cellular characteristics to those cryopreserved with 10% DMSO (Park et al., 2017).

Furthermore, the presence of FBS greatly limits the clinical application of cryopreserved cell products (Ling et al., 2022). Researchers have also developed xenofree cryopreservation solutions, demonstrating the feasibility of FBS-free cryopreservation (Mochizuki and Nakahara, 2018). Following international standards for drug manufacture, Ducret et al. (2015b) created an innovative *in vitro* technique for the separation, long-term cryopreservation, and quick expansion of DPSCs. This protocol included enzyme-free cell selection, xenofree products, and SFM. A company has invented a DPSCs cryoprotectant that is free of DMSO and FBS, avoiding the potential for animal serum contamination, reducing cell apoptosis, and enabling cells to recover their activity quickly after cryopreservation (Ying, 2021).

The potential threats posed by cryoprotectants and FBS to the safety and functional characteristics of DPSCs cannot be ignored. Currently, there are many cryopreservation products available for DPSCs. However, it is unclear whether these cryoprotective solutions can be used in clinical research, which greatly hinders the development of stem cells in the therapeutic field. Therefore, it is an important foundation for further clinical application of stem cell products to explore a universal cell cryoprotectant that is free of animal-sourced ingredients and suitable for direct clinical injection, ensuring the safety, timeliness, and scalability of stem cells in clinical applications.

### 8.2 Cooling rate

Another important aspect of cryopreservation is the cooling rate. A precise balance between the concentration of cryoprotectants and the cooling rate required for cryopreservation must be maintained (Dissanayake et al., 2010). Osmotic pressure causes cells to dry and shrink when they are cooled too slowly, while intracellular ice production may result from rapid cooling (Chen et al., 2011b; Marquez-Curtis et al., 2015). According to Woods et al. (2009), freezing DPSCs at a pace of −1°C/min in an isopropanol bath before moving them to liquid nitrogen is the best option for preserving them. However, programmed freezing of DPSCs is more complicated and requires higher equipment demands, and uncontrolled rate freezing is a convenient and promising method. DPSCs can survive and maintain their phenotypic characteristics in uncontrolled long-term freezing at −80°C (Raik et al., 2020). DPSCs subjected to uncontrolled freezing remained biologically and functionally stable after 1 year at −80°C, and this was independent of DMSO concentration (Kumar et al., 2015). According to Pilbauerova et al.'s analysis of the effects of uncontrolled rate freezing and 10% DMSO as a cryoprotectant on DPSCs stored for 6 and 12 months, DPSCs were able to withstand stressful situations without losing their stemness (Pilbauerova et al., 2021). The uncontrolled rate freezing method may be a better choice.

### 8.3 3D scaffold cryopreservation

Obtaining enough stem cells for tissue engineering applications often requires several months of time-consuming, expensive *in vitro* growth and multiplication. A well-preserved live-cell biological material structure might therefore be created, eliminating the long waiting period and cutting down on medical expenses.

The elimination of the transplantation preparation process and the ability to obtain standardized cell products as needed are two reasons why low-temperature preservation of cell-microsphere systems is thought to be crucial for clinical translation and commercialization (Gryshkov et al., 2015; Gurruchaga et al., 2018). Human DPSCs contained in gel microspheres made by Yang et al. using an electrostatic microdroplet approach were able to adhere, disseminate, multiply, produce ECM proteins, and have a tendency to inhabit the outer layer (Yang et al., 2021). Furthermore, gradual freezing might be used to typically cryopreserve cells in the GelMA microsphere system. During cryopreservation and thawing, the GelMA microspheres kept their structural integrity, and the thawed cells continued to operate normally. Umemura et al. (Umemura et al., 2011) encapsulated DPSCs in a biocompatible material consisting of alginate, CaCO_3_, and glucose-d-lactone and stored them in a cryoprotectant containing DMEM, ethylene glycol, sucrose, and polyvinylpyrrolidone. The thawed DPSCs were normal in morphology, maintained multipotency, had high proliferation ability, and expressed MSC-specific markers. 3D scaffolds for cryopreservation of DPSCs hold promising prospects for future applications.

### 8.4 Cryopreservation of whole teeth or dental pulp tissues

Especially for clinical usage, DPSCs isolation is expensive and time-consuming. Additionally, there is a chance of tissue contamination in operating rooms at hospitals and dentistry clinics since they lack sterile settings. Therefore, cryopreservation of whole teeth or isolated dental pulp tissues has practical value (Malekfar et al., 2016; Wang et al., 2022). By reducing the chance of contamination during surgery after tooth extraction and increasing the likelihood of successfully obtaining healthy DPSCs, this approach also preserves the phenotypic. Furthermore, 70% of DPSCs may be recovered from complete teeth after 1 month of cryopreservation in liquid nitrogen, and high-proliferative DPSCs can be produced from removed teeth kept in PBS at 4°C for up to 120 h (Perry et al., 2008). However, other investigations have revealed that only about 20% of viable DPSCs can be taken from diseased or healthy teeth that have been cryopreserved (Woods et al., 2009; Chen et al. (2011b). From the current evidence, direct cryopreservation of teeth does not yield good results and requires further exploration of feasible options. Moreover, the hard tissue of teeth can hinder the penetration of cryoprotectants into dental pulp, and cryopreservation of dental pulp tissue may be a better method (Takebe et al., 2017). Additionally, the application of a magnetic field during ultralow-temperature preservation can enhance the permeability of cryoprotectants, and magnetic cryopreservation may be a method suitable for the cryopreservation of intact teeth and dental pulp tissues (Lee et al., 2012b).

In recent years, several patents have been published for optimizing DPSCs, tooth, and dental Pulp storage, with varying compositions of added components, as shown in Table 8. From the table, it can be seen that hydroxyethyl starch and mannitol are commonly used cryoprotectants in current optimization schemes, and other components of cryopreservation solutions are mainly chemical substances or plant extracts.

**TABLE 8:** Representative of patents for optimization of DPSCs, teeth, and dental pulp storage.

First inventor	Status	Storage objects	Additives or method	Storage time	Storage temperature (°C)	Survival rate	Proliferation	Other	Patent
Xin Bingchang	Application	DPSCs	FBS, hydroxyethyl starch, sodium saccharin, glycerol, sodium dodecyl sulfate, D-glucose and disodium ethylenediaminetetraacetate dihydrate	6 months	−196	↑	N/A	N/A	Bingchang et al. (2021)
Zhang Junjie	Application	DPSCs	Sea algae trehalose, hydroxyethyl starch, mangiferin, evening primrose oil and capsaicin	6 months	−196	↑	↑	N/A	Junjie and Yan (2022)
Zhang Mingxin	Application	DPSCs	Glycerol, ultrafine corn cob powder, stevia extract (steviol glycosides), polysorbate 40, sodium hyaluronate and polyvinylpyrrolidone	12 months	−196	↑	N/A	N/A	Mingxin and Lei (2022)
Yang Ying	Grant	DPSCs	Titanium nanotubes, 5′-adenosine diphosphate sodium, vitamin E, 1,8-cineole and glycerol	12 months	−196	↑	N/A	No need for complex programmed cooling	Ying (2021)
Du Hongwu	Grant	DPSCs	Glycerol, glycine, mannitol, PEG, arginine, human serum albumin, sucrose solution, heparin sodium, SDS and Tween	24 h	−80	No effect	No effect	N/A	Hongwu et al. (2023)
Zhang Liang	Application	DPSCs	Chrysanthemum morifolium protein peptides, guar gum, hyaluronic acid, glucose, L-glutamine and D-mannitol	72 h	2–6	↑	N/A	N/A	Liang and Qiang (2022)
Zhang Mengyang	Grant	DPSCs	0.9% sodium chloride solution, penicillin, sodium hyaluronate, potassium dihydrogen phosphate, 2-hydroxybenzylamine, niacin ethyl ester, polyadenylic acid and vitamin K4	48 h	0–6	↑	N/A	N/A	Mengyang and Jianwei (2023)
Ding Ye	Application	DPSCs	Brown algae polysaccharide sulfate, erythritol anhydride, actinidine, sodium dihydrogen phosphate, vitamin E and physiological saline	96 h	2–8	↑	N/A	N/A	Ye and Chen (2022)
Chen Haijia	Grant	Teeth	DMSO and D-glucose	6 months	−80	↑	N/A	N/A	Haijia et al. (2018)
Ge Xiaohu	Application	Teeth	Sodium chloride, potassium chloride, disodium hydrogen phosphate, potassium dihydrogen phosphate, penicillin, streptomycin and amphotericin B	72 h	4	No effect	N/A	Inhibited bacterial proliferation	Xiaohu et al. (2017)
Silvia Gioventù	Application	Dental pulp	The teeth were perforated with a laser after removing the physiological seat from the cervical area of the tooth. Following perforation, the teeth were brought into contact with a cryoprotectant and then frozen	12 h	−50 to 90	N/A	↑	N/A	Gioventù et al. (2013)
Ye Qingsong	Application	Dental pulp	Growth factors, hormones, proteins, vitamins, reducing substances, adhesive substances, enzyme inhibitors, trace elements, human serum albumin and Cryosure DEX-4	Unspecified	−196	N/A	↑	The time for tissue block cells to crawl out was shorter	Qingsong et al. (2019a)

DPSCs, dental pulp stem cells; SFM, Serum-free medium; DMSO, dimethyl sulfoxide; FBS, fetal bovine serum; PEG, polyethylene glycol; SDS, sodium dodecyl sulfate. Note: The list of patents included in this table is not the product of a systematic search but is filtered for relevance to the subject.

In addition, factors such as freezing temperature, storage density, and storage method also affect the cryopreservation of DPSCs. Cryopreservation of stem cells offers several advantages for clinical use, including flexible treatment planning, improved product quality, and the potential for large-scale cell production (Lee et al., 2018). Furthermore, low-temperature storage can help maintain the genetic stability of cell strains and enable the non-continuous use of cells. However, additional experimental data and further research and clinical trials are needed to ensure the effectiveness and safety of this approach.

## 9 Recovery of DPSCs

The assessment of the survival ability of thawed stem cells is crucial for clinical transplantation. Thawed stem cells often undergo apoptosis due to the activation of the caspase pathway (Desoutter et al., 2019), resulting in poor survival, proliferation, antioxidant capacity, and pluripotency. This may be due to changes in the spatial configuration of cell membrane proteins during the cryopreservation process (Desoutter et al., 2019; Dimas-Gonzalez et al., 2019). Thawing time, washing medium, temperature, centrifugation time, and centrifugation force can all affect the recovery of cells after cryopreservation (Honge et al., 2017). Rapid thawing is generally believed to reduce the chance of damage caused by local re-freezing of cells (Baboo et al., 2019).

However, there are few studies and patents focused on optimizing the recovery methods for DPSCs. Recombinant human bFGF is a type of peptide that can enhance cell proliferation and prolong cell lifespan. According to Luo et al.'s research, 20ng/mL of bFGF can dramatically boost the proliferation of thawed DPSCs by blocking apoptosis, activating the ERK pathway, up-regulating TRPC1, and other mechanisms (Luo et al., 2021). This proliferative advantage can be passed on to subsequent passages while maintaining stemness and pluripotency. Zhang et al. (Jianqiu and Xinhong, 2022) added phospholipids, musk ketone, and irisin to a basal culture medium to create a revival solution that promotes high post-thaw viability and good proliferative activity of cryopreserved DPSCs, allowing for direct use in routine expansion culture. A biotechnology company invented a DPSCs revival medium by adding EGF, transferrin, insulin, α-asarone, N-acetyl-5-methoxytryptamine, corn peptide, casein phosphopeptide, sodium cholate, citric acid zinc, and citronellol to a basal culture medium. Using this revival medium, DPSCs can be rapidly revitalized and easily expanded, facilitating clinical research (Shuang and Jie, 2022). The initial step in using DPSCs in therapeutic settings is to extract them from a cryopreserved cell bank. The effectiveness of the treatments is directly impacted by the quality of the retrieved cells. Therefore, researchers should put this aspect as one of the research priorities.

## 10 Discussion

This article provides a detailed analysis of the optimization research data from the extraction to the cryopreservation process of DPSCs. Based on current evidence, the enzyme-free tissue block culture method is more compliant with international regulatory requirements for primary extraction of DPSCs, although more research is needed to overcome its limitations. Serum-free culture methods, which do not use animal serum, are safer and more suitable for large-scale expansion of DPSCs *in vitro*. However, the formula for SFM is diverse, and further efforts are needed to standardize it. 3D culture, which is closer to the physiological environment of cells, is more suitable for the growth and expansion of DPSCs. Scaffold culture and scaffold-free culture each have their characteristics and applications. In addition, hypoxic culture, pathway regulation, and cell immortalization all have research and application value and can be used to fully exploit the properties of DPSCs. Regarding the cryopreservation of DPSCs, the safety for clinical application can be improved by reducing the concentration of DMSO in the cryopreservation medium and omitting the addition of FBS. Consistent with SFM, the existing formulas for cryopreservation solutions are diverse, and there is still a long way to go to standardize and normalize them. In addition, an uncontrolled rate freezing method is more convenient, and 3D scaffold cryopreservation can meet clinical emergency needs, while direct freezing of teeth or dental pulp tissue is more suitable for primary medical institutions. Finally, growth factors or other additives can be added to improve the resuscitation effect and maintain the biological characteristics of DPSCs during recovery. In addition to the extraction, culture, freezing, and recovery of DPSCs mentioned above, other factors can affect the efficiency and safety of DPSCs *in vitro* culture, such as donor age, tooth transportation, short-term storage, immunophenotyping and selection of DPSCs, and culture dish coating.

To realize clinical applications, stem cell therapy should transition from the experimental level in the laboratory to the clinical level, which requires following a series of standards and procedures throughout the manufacturing process. It is also necessary to ensure that the raw materials and equipment used meet regulatory requirements, and have undergone sufficient quality control and validation to ensure that the produced DPSCs meet quality standards and are safe and reliable for clinical use. EU GMP has strict requirements on all aspects of product production, including quality management systems, personnel training, facilities and equipment, raw materials and reagents, process control and documentation. These may be the main obstacles that limit the application of DPSCs to clinical trials, along with the poor translation rate from laboratory to clinic. Although the techniques for isolating, amplifying, and preserving DPSCs have undergone significant advancements, more study is required to create improved techniques and optimized protocols that adhere to standardization requirements, maintain stem cell properties over time, minimize phenotypic variation, and address safety concerns associated with using DPSCs in clinical treatments. There is still much work to be done before DPSCs may be applied to clinical therapies. To solve these restrictions, researchers, physicians, and regulatory organizations must work together to create innovative approaches. It is essential to explore the potential of DPSCs in effective clinical treatments and in improving patients’ wellbeing.
